# Cytotoxic Lesions of the Corpus Callosum (CLOCCs) Related to Hypoglycemia: Clinical Insights and Pathogenesis

**DOI:** 10.7759/cureus.69965

**Published:** 2024-09-23

**Authors:** Koji Hayashi, Yuka Nakaya, Midori Ueda, Maho Hayashi, Shin-ichiro Azuma, Asuka Suzuki, Mamiko Sato, Yasutaka Kobayashi

**Affiliations:** 1 Department of Rehabilitation Medicine, Fukui General Hospital, Fukui, JPN; 2 Department of Internal Medicine, Fukui General Hospital, Fukui, JPN; 3 Graduate School of Health Science, Fukui Health Science University, Fukui, JPN

**Keywords:** cytotoxic lesions of the corpus callosum, hypoglycemia, mild encephalitis/encephalopathy with a reversible isolated splenial lesion, reversible splenial lesions and reversible splenial lesion syndrome, splenium of the corpus callosum

## Abstract

We describe the case of an 88-year-old man with cytotoxic lesions of the corpus callosum (CLOCCs) related to hypoglycemia. The patient developed a disturbance of consciousness following excessive alcohol consumption and anorexia. In the emergency room, his blood sugar level was 9 mg/dL, and he was immediately treated with a rapid infusion of glucose. Brain magnetic resonance imaging (MRI) revealed hyperintensity in the corpus callosum and bilateral deep white matter on diffusion-weighted imaging, along with decreased apparent diffusion coefficient values. The following day, these findings were attenuated, and he was diagnosed with CLOCCs. In this report, we discuss clinical insights and the possible pathogenesis of the development of CLOCCs due to hypoglycemia, considering the previous literature.

## Introduction

Hypoglycemia can cause neurological symptoms such as behavioral changes, confusion, fatigue, seizures, coma, and if left untreated, potentially death [1]. In terms of the causes of hypoglycemia, it is rare in individuals without diabetes, but when it does occur, common causes include medications, alcohol, severe illness, counter-regulatory hormone deficiencies, and non-islet cell tumors [1]. Since hypoglycemia is typically corrected quickly and symptoms like impaired consciousness often improve rapidly, brain imaging is not frequently performed in the emergency room (ER), even when patients visit the hospital.

Cytotoxic lesions of the corpus callosum (CLOCCs) are an abnormal condition mainly in the splenium of the corpus callosum (SCC), which can be detected by brain magnetic resonance imaging (MRI) [1]. Due to diffusion restriction by apparent diffusion coefficient (ADC) images, the corpus callosum lesions are considered cytotoxic lesions [2]. The term for these corpus callosum lesions varies in the literature, including transient lesions of the SCC, mild encephalitis/encephalopathy with reversible splenial lesions (MERS), reversible splenial lesions, and reversible splenial lesion syndrome (RESLES) [2]. In recent years, CLOCCs have come to be used as a term that better reflects the pathology assumed by radiological findings. CLOCCs are associated with various diseases including infection, seizures/status epilepticus, drug therapy, alcohol, metabolic disturbance, subarachnoid hemorrhage, trauma, and malignancy, among others [3]. However, the reports of CLOCCs caused by hypoglycemia are limited. In this report, we describe a case of CLOCCs related to hypoglycemia and mention the clinical insights and pathogenesis.

## Case presentation

An 88-year-old man with a history of prostate cancer, rheumatoid arthritis, dementia, and multiple fractures, including those of the femur and ribs, developed a disturbance of consciousness and was transported to our hospital. He was not on any medication. Two days before onset, he had consumed excessive alcohol. The day before onset, he was still able to eat dinner. Vital signs included a heart rate of 109 beats per minute, blood pressure of 147 mmHg, and peripheral oxygen saturation (SpO2) of 100% on 5L/minute of oxygen. The patient had altered consciousness, with a Glasgow Coma Scale score of E1V1M1. The pupils were equal in diameter at 3.0 mm bilaterally, and the light reflex was brisk on both sides. Blood tests revealed significant decreases in hemoglobin, albumin, and cholinesterase, as well as elevated levels of urea nitrogen, uric acid, aspartate aminotransferase, alkaline phosphatase, lactate dehydrogenase, γ-glutamyl transferase, amylase, sodium, C-reactive protein, D-dimmer, and lactate acid (Table 1).

**Table 1:** The results of blood tests on admission.

Inspection item	Result	Reference range
White blood cell count	7100 /μl	(3300–8600)
Red blood cell count	307×10⁴ /μl	(435–555×10⁴)
Hemoglobin	12.0 g/dl	(13.7–16.8)
Blood platelet	19.7×10⁴ /μl	(15.8–34.8)
Glucose	9 mg/dl	(73–109)
Hemoglobin A1c	4.9%	(<5.5%)
Total protein	6.7 g/dl	(6.6–8.1)
Albumin	3.8 g/dl	(4.1–5.1)
Blood urea nitrogen	22.4 mg/dl	(8.0–20.0)
Creatinine	1.05 mg/dl	(0.46–0.79)
Uric acid	11.5 mg/dl	(3.7–7.0)
Ammonia	33 μg/dl	(12–70)
Total bilirubin	0.6 mg/dl	(0.4-1.2)
Alkaline phosphatase	497 U/l	(106–322)
Aspartate aminotransferase	124 U/l	(13–30)
Alanine aminotransferase	23 U/l	(7–30)
Lactate dehydrogenase	349 U/l	(124–222)
Creatine kinase	189 U/l	(41–153)
γ-glutamyltransferase	131 U/l	(13-64)
Amylase	751 U/l	(44–132)
Choline esterase	201 U/l	(240-486)
Sodium	148 mmol/l	(138–145)
Potassium	4.2 mmol/l	(3.6–4.8)
Chlorine	105 mmol/l	(101–108)
C-reactive protein	0.6 mg/dl	(0.00–0.14)
Lactate acid	108.9 mg/dl	(4.0–16.0)
Prothrombin time	10.8 sec	(9.6–13.1)
Prothrombin time and international normalized ratio	0.99	(0.8-1.2)
Activated partial thromboplastin time	25.7 sec	(24-34)
D-dimer	13.3 μg/ml	(21.0–29.0)

**Figure 1:**
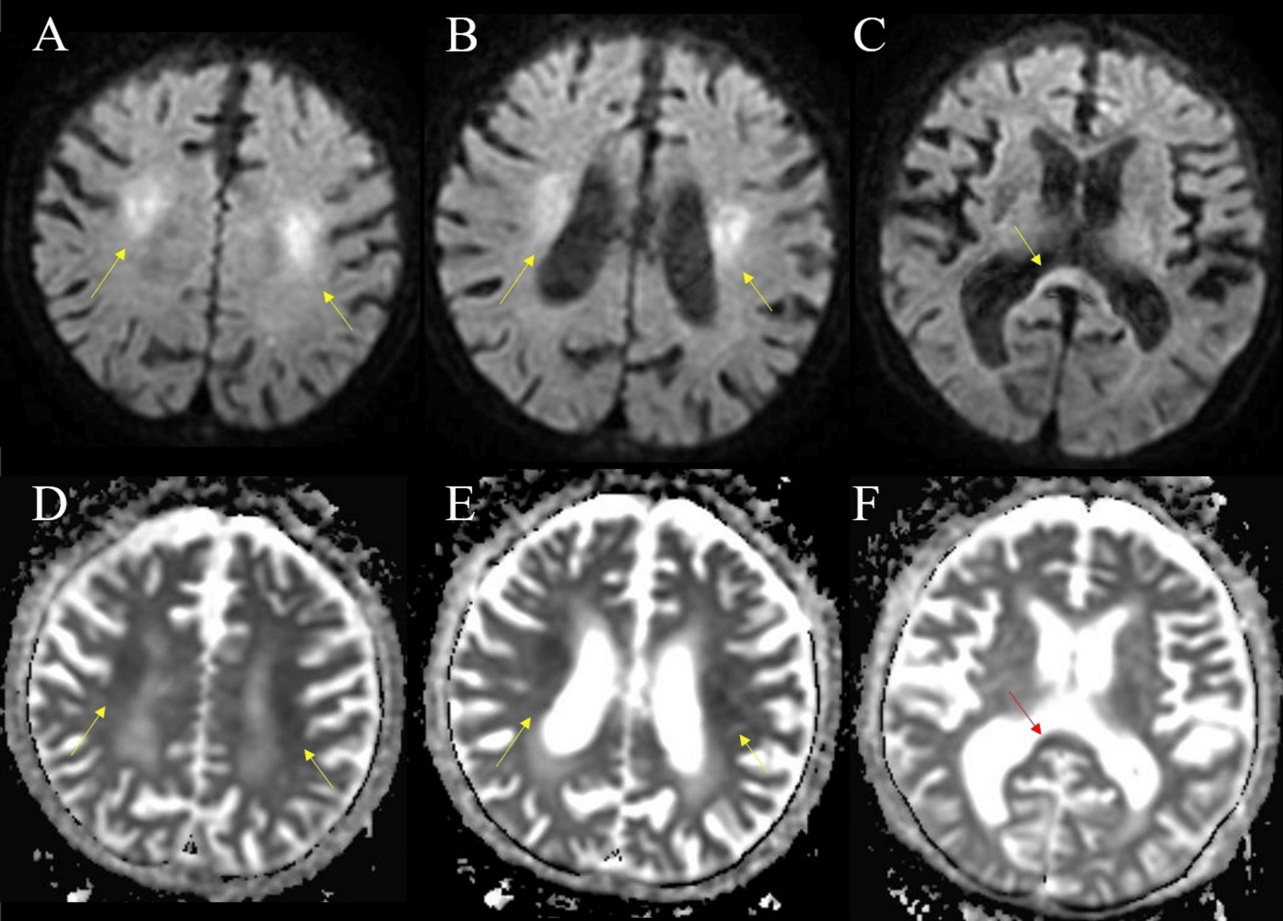
The results of brain magnetic resonance imaging (MRI) on admission. (A-C) Diffusion-weighted brain MRI showing hyperintensity in the splenium of the corpus callosum (SCC) and the bilateral deep white matter symmetrically (arrowheads). (D-F) Apparent diffusion coefficient (ADC) values were decreased in the areas of hyperintensity by diffusion-weighted brain MRI (arrowheads).

**Figure 2:**
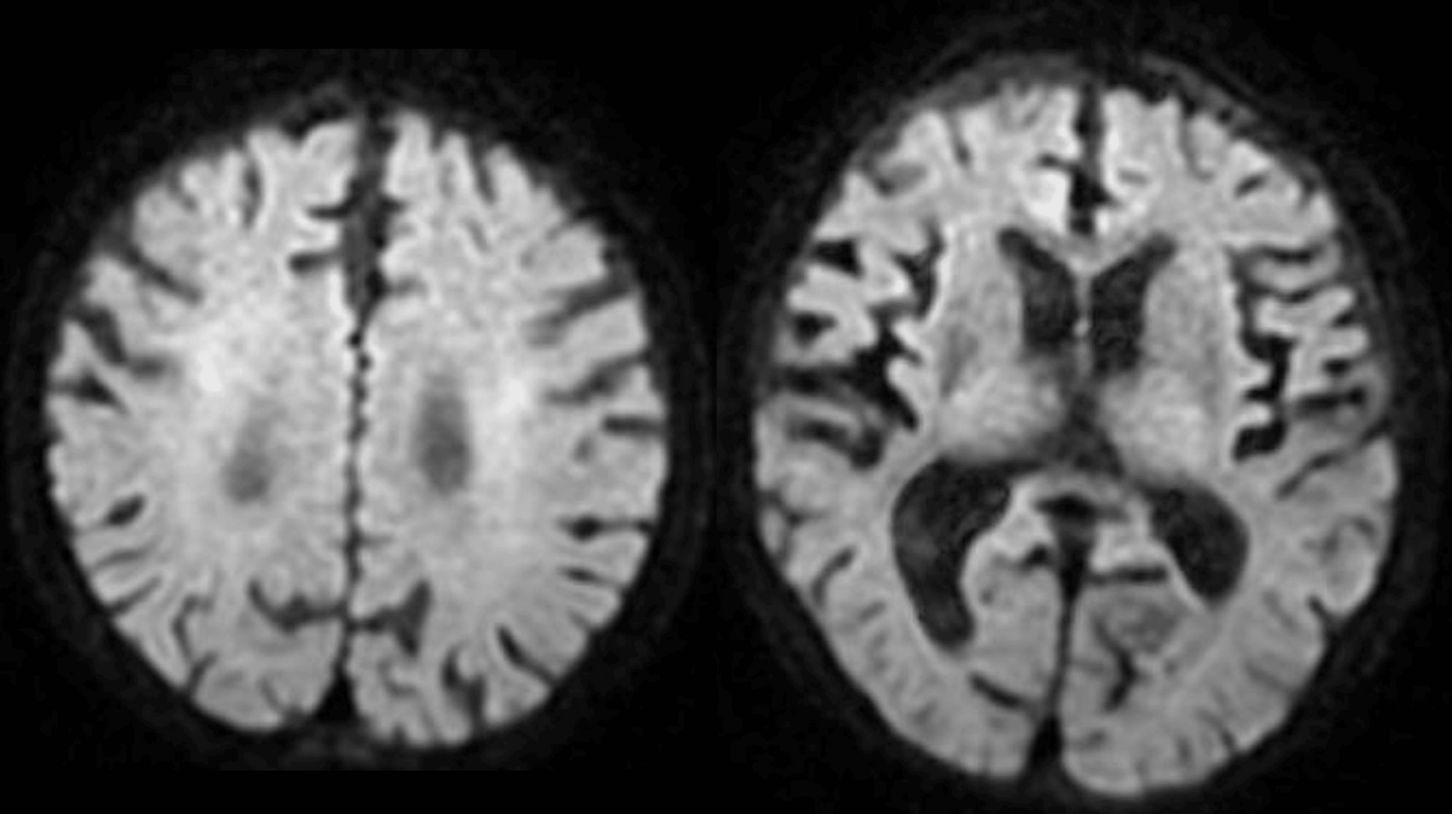
The results of diffusion-weighted brain magnetic resonance imaging (MRI) on day two of admission. Diffusion-weighted brain MRI showing attenuation of the areas of hyperintensities noted on admission.

A blood gas analysis revealed severe metabolic acidosis. In the emergency room, his blood sugar level was critically low at 9 mg/dL, and he was immediately treated with a rapid infusion of glucose (50% glucose, 40 mL). Additionally, multiple vitamins, including thiamine, were administered. Thirty minutes after the infusion, his serum glucose level had increased to 207 mg/dL. Diffusion-weighted brain MRI showed hyperintensities in SCC and bilateral deep white matter near the lateral ventricle and ADC values were decreased in the areas of hyperintensity by diffusion-weighted MRI (Figure 1A-1F). Although he was treated with glucose infusion, vitamin B1 and B12 supplementation, and extracellular fluid infusion, his consciousness did not return on the first day of admission but was regained the following day. His orientation also improved, and he was able to accurately answer questions regarding his age, location, and time. However, due to pre-existing dementia, some incoherent conversations were observed, and he occasionally failed to follow instructions. There was no obvious paralysis, and he was able to eat independently. A re-study of the brain MRI on day two of admission showed attenuation of abnormal intensity (Figure 2). He was treated with rehabilitation therapy and discharged on day 95 without any sequelae.

## Discussion

We present a case of CLOCCs related to hypoglycemia. On admission, blood tests revealed significant hypoglycemia and mildly elevated serum sodium levels. Diffusion-weighted brain MRI revealed hyperintensities in the corpus callosum and bilateral deep white matter. The ADC value in the areas of hyperintensity seen on diffusion-weighted images was decreased. These findings were attenuated on the next day of admission. Based on the radiological characteristics, this case was diagnosed as typical CLOCCs. Although it took time with rehabilitation treatment, he was able to be discharged without any lasting aftereffects.

Before the publication of pathological findings related to CLOCCs, the etiology was assumed based on the radiological characteristics including diffusion restriction by the ADC value, indicative of cytotoxic edema [2]. It has been widely hypothesized that an inflammatory process involving cytokines triggers the accumulation of glutamate in the extracellular space, leading to cytotoxic edema, particularly affecting astrocytes [4]. Additionally, Tada et al. speculated that the condition may be caused by intramyelinic edema and infiltration of inflammatory cells [5]. In 2024, our research group reported brain pathology in an autopsy case of CLOCCs due to hypoglycemia [6]. Like this case, the previous autopsy case had lesions in both SCC and deep white matter, which is classified into type II; type I is where the lesions are confined to the splenium of the corpus callosum, and type II is where the lesions extend to include both the splenium and subcortical or deep white matter [7]. Type II typically has a longer clinical course and is more likely to result in neurological sequelae [7]. Pathological analysis proved that intramyelinic edema, myelin pallor, microglial reactions, and loss of fibrous astrocytes in both SCC and deep white matter lesions as well as minimal infiltration of lymphocytes into the SCC were noted [6]. In addition, demyelination was suspected because of the preservation of axons in myelin pallor [6]. Thus, our group concluded that transient demyelination following cytotoxic edema leads to CLOCCs.

Although CLOCCs associated with hypoglycemia have been previously reported [6,8,9], they are not a common brain finding in cases of hypoglycemia. Kang et al. described a series of 11 cases with diffusion-weighted MRI findings in hypoglycemic encephalopathy, where many cases showed hyperintensities in the corona radiata; however, none exhibited abnormal signals in the corpus callosum [10]. In another study, Katoh et al. reported that only four out of seventy cases (5.7%) of hypoglycemia had reversible splenial lesions on diffusion-weighted brain MRI [11]. When glucose levels in the brain drop below 1 mM (18 mg/dL) for a certain period, there is a massive release of the excitatory amino acid aspartate into the brain's limited extracellular space, overwhelming the excitatory amino acid receptors on neuronal dendrites [12]. It is thought that the corpus callosum is predominantly affected due to the high density of oligodendrocytes with a large number of glutamate-sensitive receptors [2]. However, the simultaneous damage to the deep white matter remains unexplained.

One of the potential triggers of CLOCCs may be the serum sodium level [13]. As well as CLOCCs, central pontine myelinolysis (CPM) is a well-known CNS disease causing demyelination in the brain. This clinical condition is characterized by symmetrical non-inflammatory demyelinating lesions localized to the basilar pons. Adams et al. first described four cases of CPM associated with chronic alcoholism and malnutrition in 1959 [14]. Since then, numerous reports on CPM have been published, with rapid correction of hyponatremia identified as a primary cause, as demonstrated in animal studies [15-18]. However, it has also been reported that not only rapid correction of hyponatremia but also rapid correction of hypernatremia can lead to CPM [19]. Additionally, a case of CPM following the initiation of hemodialysis has been documented [20]. Based on these reports, it appears that the pathogenesis of CPM is related not to serum sodium levels per se but to sudden changes in osmotic pressure. Both serum glucose and sodium play crucial roles in regulating serum osmolality. The patient may have developed CLOCCs as a result of experiencing hypoglycemia due to some sudden cause. Alternatively, while treating hypoglycemia in the ER is a standard medical procedure, the change in serum osmolality that resulted from this treatment might have led to demyelination of the corpus callosum, which in turn could have caused the occurrence of CLOCCs. It is unclear which of these is the cause, but it is possible that changes in serum glucose levels and sodium abnormalities, which induce changes in osmolality, may contribute to the development of CLOCCs.

## Conclusions

We describe a case of CLOCCs related to hypoglycemia. Furthermore, we present its pathogenesis based on the previous pathological report from our research group. CLOCCs related to hypoglycemia may be caused by rapid changes in plasma osmolarity and resulting demyelination because of the presence of abnormalities in plasma sodium. Further studies are needed to reveal the underlying mechanism of CLOCCs related to hypoglycemia.
